# Comparison of Oral and Intranasal Midazolam Premedication in Children Undergoing Cleft Lip and Palate Surgery: A Randomized Controlled Trial

**DOI:** 10.7759/cureus.93437

**Published:** 2025-09-28

**Authors:** Mayumi Hashimoto, Aiji Sato-Boku, Naoko Tachi, Izumi Kuroda, Shota Tsukimoto, Takuro Sanuki, Atsushi Abe

**Affiliations:** 1 Department of Anesthesiology, Aichi Gakuin University, Nagoya, JPN; 2 Department of Dental Anesthesiology, Nagasaki University Hospital, Nagasaki, JPN; 3 Department of Clinical Physiology, Medical and Dental Sciences, Graduate School of Biomedical Sciences, Nagasaki University, Nagasaki, JPN; 4 Department of Oral and Maxillofacial Surgery, School of Dentistry, Aichi Gakuin University, Nagoya, JPN

**Keywords:** cleft lip and palate, intranasal administration, midazolam, pediatric anesthesia, premedication

## Abstract

Introduction: Preoperative anxiety in pediatric patients undergoing surgery may result in postoperative maladaptive behaviors and prolonged recovery. Midazolam is a commonly used premedication for anxiety reduction. However, the optimal administration route remains unclear, particularly in patients requiring repeated surgeries, such as those with cleft lip and/or palate.

Methods: We conducted a prospective, randomized controlled trial including 68 pediatric patients aged 1-10 years scheduled for cleft lip and/or palate surgery. The patients were randomly assigned to receive midazolam either orally (0.5 mg/kg in grape juice) or intranasally (0.3 mg/kg via spray). The primary endpoints were emotional reaction during drug administration (Facial Hedonic Score) and ease of administration (Ease of Administration Score). The secondary outcomes included sedation score, ease of parental separation, mask acceptance, and safety parameters. Statistical analysis was conducted using the Mann-Whitney U test with a significance level of *p* < 0.05.

Results: The oral administration group exhibited significantly lower Facial Hedonic Scores than the intranasal group, suggesting less emotional distress (p = 0.002). No significant differences in ease of administration, sedation depth, parental separation, or mask acceptance were observed between the groups. Furthermore, no adverse events, including respiratory depression and laboratory abnormalities, occurred in either group.

Conclusion: Midazolam oral administration is associated with enhanced emotional tolerance in pediatric patients undergoing cleft-related surgeries compared with intranasal administration, with similar efficacy and safety profiles. This route may be preferable in younger or anxious children to minimize preoperative stress.

## Introduction

Cleft lip and palate are congenital malformations resulting from incomplete fusion of facial structures during fetal development and are classified into cleft lip, palate, and jaw as well as their associated complications [1]. This condition has a relatively high incidence worldwide, occurring in approximately 1 in 500-700 births, making it one of the most common congenital anomalies [2]. Moreover, it requires long-term, comprehensive intervention from the neonatal period through adolescence by a multidisciplinary team in collaboration with plastic and oral surgeons, orthodontists, pediatricians, and speech therapists. Surgical treatment is often performed under general anesthesia in multiple sessions, which may pose considerable psychological stress on pediatric patients. This perioperative stress has been reported to result in postoperative abnormal behavior and emotional distress, and premedication with benzodiazepine-derived drugs, such as midazolam, is widely adopted as a preventive measure [3].

Midazolam premedication is commonly used to reduce preoperative anxiety in children. Although intramuscular injection was historically adopted, it is now largely avoided due to the pain and distress it causes. Consequently, non-invasive routes such as oral and intranasal administration have become more prevalent in clinical practice. However, each of these alternative routes presents its own challenges, highlighting the need to determine the most appropriate method for pediatric patients [4]. Oral administration is simple and noninvasive. In some regions, flavored oral syrup formulations are available, and bitterness is not a major issue. However, in Japan, where only the IV/IM formulation is available for off-label oral use, the inherent bitterness of midazolam can pose challenges, such as refusal to take the medication and incomplete administration. Meanwhile, intranasal administration offers a rapid onset of action; however, irritation and fear of nasal sprays may reduce acceptability. Moreover, children with cleft lip and palate often undergo multiple surgeries as they grow, and their preoperative anxiety and stress may be more pronounced than those of other pediatric patients. However, studies directly comparing the acceptability, efficacy, and safety of oral and intranasal administrations in this special pediatric population are scarce. Therefore, elucidating the most appropriate administration route for preanesthetic medication, a decision often made under clinical constraints, that is effective, safe, and less painful for patients, is considered to have significant practical and clinical implications.

This study aimed to compare the efficacy and tolerability of oral and intranasal administrations of midazolam as premedication for anesthesia in pediatric patients undergoing cleft lip and palate surgery. The study included patients aged 1-10 years who received midazolam either orally (mixed with grape juice) or intranasally (as a spray of the undiluted or diluted solution). The patients’ responses during administration, ease of administration, sedation status, mother-infant separation status, and mask acceptance during anesthesia induction were evaluated using multiple assessment criteria. In addition, the occurrence of safety-related adverse effects was examined. This study was conducted as a superiority-type comparison to explore whether one route would provide more favorable acceptance and sedation outcomes. This study aimed to facilitate the determination of the optimal administration route for premedication aimed at reducing preoperative anxiety.

## Materials and methods

Ethical considerations and trial registration

This study was conducted in accordance with the ethical principles of the Declaration of Helsinki (1964 and subsequent revisions). Before the study initiation, approval from the Ethics Committee of the School of Dentistry, Aichi Gakuin University, was obtained (approval no. 538). The study was registered in the UMIN Clinical Trials Registry (UMIN000032540) in accordance with Japan’s clinical trial registration system. The original IRB approval document in Japanese, issued on the official letterhead of Aichi Gakuin University School of Dentistry, has been submitted along with its English translation. Informed consent was obtained from the trial participants’ legal representatives based on written explanations and voluntary agreement. For participants capable of communication, informed assent was obtained using explanatory documents written in simple language appropriate for their age, to the extent possible.

Study design and population

**Figure 1:**
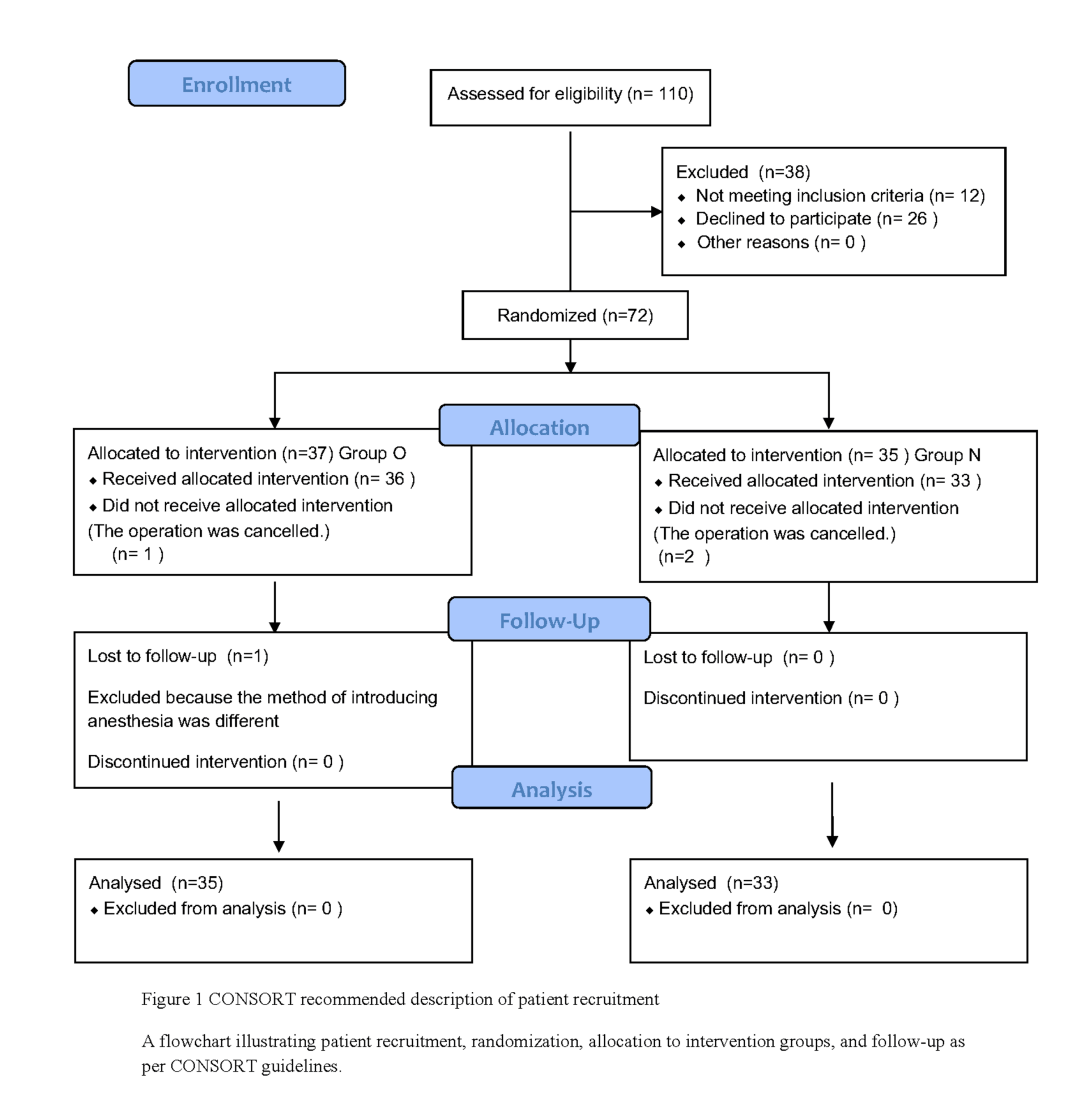
CONSORT recommended description of patient recruitment A flowchart illustrating patient recruitment, randomization, allocation to intervention groups, and follow-up as per CONSORT guidelines. CONSORT: Consolidated Standards of Reporting Trials

This study was conducted as a prospective randomized controlled trial. Due to the nature of the interventions, neither the participants nor the investigators could be blinded to the assigned administration route. A total of 110 pediatric patients aged 1-10 years who were scheduled to undergo cleft lip and palate repair or pharyngeal flap surgery at our hospital were screened from March 25, 2019. The inclusion criteria were hematological tests (WBC, platelet count, and Hb) and blood biochemical tests (AST, ALT, TB, ALP, LDH, Cr, and CK) conducted within one week postoperatively, with results that were within the facility’s reference ranges. The exclusion criteria included (i) patients with severe heart disease, liver dysfunction, or kidney dysfunction; (ii) patients with a history of allergy or severe hypersensitivity to midazolam; and (iii) patients considered ineligible to participate in the study by the attending physician. Of the 110 patients enrolled, 26 refused participation, and 12 were excluded due to failure to meet the criteria. The remaining 72 patients were randomly allocated to the oral administration group (n = 35) and intranasal administration group (n = 37) using a computer-generated random number table by a research coordinator not involved in anesthesia or outcome assessment. Group assignments were concealed in sequentially numbered, sealed opaque envelopes, which were opened by the anesthesiologist immediately before drug administration. Due to the nature of the interventions, blinding of participants, investigators, and outcome assessors was not feasible. Ultimately, 35 and 33 patients in the oral and intranasal administration groups, respectively, were analyzed (Figure 1).

Administration method and anesthesia procedures

**Figure 2:**
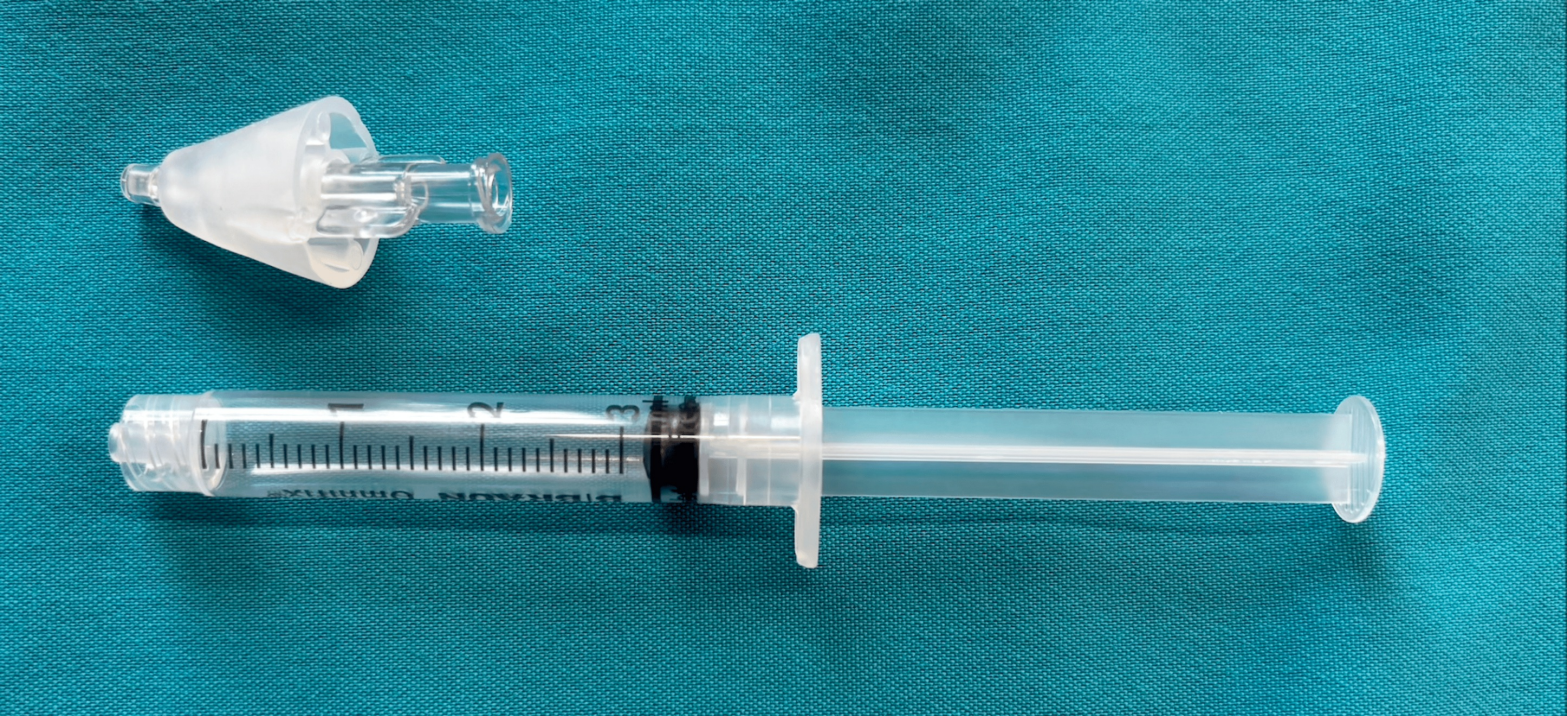
Nasal spray device used for intranasal administration of midazolam The intravenous formulation of midazolam was diluted with normal saline to a total volume of 2 mL and administered intranasally using a nasal spray device commonly used in otolaryngology (Fine Atomizer [Nasal], Yoshikawa Kasei Co., Japan). The device attaches to a syringe and delivers the solution as a fine mist suitable for intranasal application.

Midazolam premedication was administered by an anesthesiologist approximately 30 minutes before entry into the operating room. Although intranasal administration achieves more rapid absorption, a uniform interval of 30 minutes was applied to both groups to standardize the protocol and ensure comparability. For the oral administration group, midazolam was administered at a dose of 0.5 mg/kg, using the intravenous formulation (10 mg/2 mL; 5 mg/mL), mixed with grape juice to a total volume of 10 mL. At the time this study was conducted, no commercially available pediatric oral syrup formulation of midazolam was available in Japan, and therefore, the intravenous formulation was diluted with fruit juice to improve palatability. For the intranasal administration group, midazolam was administered at a dose of 0.3 mg/kg, using the intravenous formulation (10 mg/2 mL; 5 mg/mL), diluted with normal saline to a total volume of 2 mL, since no commercial preparation specifically for intranasal use was available in Japan at the time of the study. Diluted midazolam was administered via a nasal spray device used in otolaryngology (Fine Atomizer [Nasal], Yoshikawa Kasei Co., Japan) with 1 mL sprayed into each nostril (Figure 2). For both groups, the maximum dose of midazolam was 10 mg. During the 30 minutes after premedication and before induction, patients were continuously monitored with pulse oximetry, and their respiratory status was visually assessed by anesthesiologists. Continuous measurements of heart rate, respiratory rate, and blood pressure were not recorded during this interval.

General anesthesia was consistently administered to all subjects according to the following procedure. Pre-operative fasting was performed according to standard pediatric anesthesia guidelines: no solid food or milk for at least six hours, no breast milk for four hours, and no clear fluids for two hours before induction. After slow induction with oxygen and sevoflurane at 8%, 0.6 mg/kg rocuronium and 0.5-µg/kg/min remifentanil were administered, followed by endotracheal intubation. During surgery, anesthesia was maintained with sevoflurane (2%-3%), and remifentanil and rocuronium were administered as needed. Immediately before the end of surgery, 1-µg/kg fentanyl was administered, followed by 2-mg/kg sugammadex and 10-mg/kg acetaminophen suppository after the procedure.

Primary and secondary outcome measures

Outcome assessments, including the Facial Hedonic Score and sedation scales, were performed by anesthesiologists involved in perioperative patient management. Because these assessments required direct observation during drug administration, blinding of outcome assessors to group allocation was not feasible. To reduce potential bias, data entry and statistical analysis were conducted by investigators who were not involved in drug administration.

Primary endpoints

The primary endpoints of this study are as follows: (i) Patient response at the time of medication administration was evaluated using a 7-point Facial Hedonic Score, adapted from Thompson et al. [5], where 1 = very unpleasant, 2 = unpleasant, 3 = slightly unpleasant, 4 = neutral, 5 = slightly pleasant, 6 = pleasant, and 7 = very pleasant. (ii) Acceptability of drug administration (ease of administration). The ease of administration was evaluated using a modified 6-point scale (modified Ease of Administration Score), adapted from previously reported assessment tools in pediatric sedation studies, although no single validated source could be identified. The scale was defined as 1, indicating accepted readily; 2, accepted with a grimace; 3, accepted with verbal complaint; 4, accepted with a cry; 5, accepted with partial loss of drug; and 6, rejected entirely.

Secondary evaluation items

The following three items were set as secondary evaluation items, and scores were assigned at each stage from administration to induction of anesthesia: (i) Assessment of sedation status: Sedation Score (5-point scale by Wilton et al.) was used to determine the level from 1 (agitated) to 5 (asleep) [6]. Sedation status was assessed using the Sedation Score at 30 minutes after drug administration, immediately before entry into the operating room, in both groups. (ii) Ease of mother-infant separation upon entry into the operating room: Evaluated using the Parental Separation Score (Wilton et al.), ranging from 1 (easy separation) to 4 (crying and clinging to parent) [6]. (iii) Mask acceptance at anesthesia induction: The Mask acceptance score (Wilton et al.) was used, with scores ranging from 1 (excellent) to 4 (poor) [6].

Safety evaluation

Safety evaluation related to midazolam premedication was performed from the following perspectives: (i) presence of respiratory depression, evaluated via SpO₂ monitoring before and during surgery; (ii) clinical symptoms, such as anaphylactic shock, cardiovascular abnormalities, and delayed awakening, confirmed by anesthesiologists based on intra- and postoperative clinical observations; and (iii) liver and kidney function impairment, evaluated based on results of blood tests (AST, ALT, Cr, BUN, etc.) conducted the day after premedication.

Statistical analysis

Based on an effect size of 0.59 for the primary endpoint, a Type I error (α) of 0.05, and a power (1-β) of 0.80, the minimum required sample size was calculated to be 60 cases. This effect size was calculated based on the results of a pilot study (involving 10 patients in the oral administration group and 10 in the nasal administration group) conducted prior to the study. To prevent a reduction in statistical power, an expected dropout rate of 5% was considered and the final required sample size was calculated to be 68 subjects using the following formula:

Nd = N/(1 − R)² (Nd: required sample size after adjustment for dropouts, N: sample size without dropouts, R: dropout rate) [7].

Statistical analysis was conducted using the Mann-Whitney U test for continuous or ordinal variables and the chi-square test for categorical variables, with P < 0.05 considered to indicate statistical significance.

## Results

Comparison of background factors

The mean age of the subjects was 5.7 ± 3.2 years in the oral administration group and 7.2 ± 3.1 years in the intranasal administration group, with the intranasal administration group demonstrating a significantly higher mean age (P = 0.036).

As regards sex distribution, there were 21 male patients and 14 female patients in the oral administration group and 18 male patients and 15 female patients in the intranasal administration group, with no significant difference between the groups.

As regards physical characteristics, the mean height was 108.8 ± 22.4 cm in the oral administration group and 118.5 ± 21.3 cm in the intranasal administration group, whereas the mean weights were 20.0 ± 8.7 kg and 24.1 ± 10.6 kg, respectively. No statistically significant differences were observed in either parameter (Table 1).

**Table 1:** Demographic and clinical characteristics of patients This table summarizes baseline characteristics of participants in both oral and intranasal administration groups, including age, sex, height, weight, and clinical parameters. Values are presented as mean ± SD or number of patients. Statistical analysis was performed using the Mann–Whitney U test for continuous variables and the chi-square test for categorical variables; P < 0.05 was considered statistically significant.

		Oral		Nasal		P-value	
								Age (years)		5.7±3.2		7.2±3.1		0.036	
Gender (male/female)		21/14		18/15		0.699									
								Height (cm)		108±22.4		118.5±21.3		0.057	
Weight (kg)		20.0±8.7		24.1±10.6		0.113									

Some differences in terms of surgical procedures were observed between the groups, with no statistically significant differences (Table 2).

**Table 2:** Comparison of surgical procedures between the oral and intranasal administration groups Breakdown of the types of cleft lip and palate surgeries conducted across both study groups. Values are presented as number of patients. Statistical analysis was performed using the chi-square test; P < 0.05 was considered statistically significant.

		Oral		Nasal		P-value	
								Labioplasty		1		0		0.98	
Palatoplasty		11		7											
						Alveolar cleft secondary bone grafting (iliac bone)		8		16					
Alveolar cleft secondary bone grafting (tibia bone)		1		2											
						Cleft lip secondary correction		13		6					
Phatyngeal flap		0		2											
						Hard palate closure palatoplasty		1		0					

Results of primary endpoints

Patients’ Responses at the Time of Medication Administration (Facial Hedonic Score)

Statistical analysis revealed that the intranasal administration group had significantly lower Facial Hedonic Scores (worse response) than the oral administration group, with a significant difference observed between the groups (P = 0.002) (Table 3).

**Table 3:** Patients’ responses at the time of medication administration (Facial Hedonic Score) Distribution of scores measuring patients' emotional responses to drug administration using a 7-point scale: 1 = very unpleasant, 2 = unpleasant, 3 = slightly unpleasant, 4 = neutral, 5 = slightly pleasant, 6 = pleasant, and 7 = very pleasant. Values are presented as number of patients. Statistical analysis was performed using the Mann–Whitney U test; p < 0.05 was considered statistically significant.

Score		Oral		Nasal		P-value	
								1		1		4		0.002	
2		1		7											
						3		4		5					
4		6		8											
						5		14		4					
6		4		2											
						7		5		3					
Total		35		33											

Ease of Administration Score

No statistically significant difference in ease of administration was observed between the groups (P = 0.246) (Table 4).

**Table 4:** Ease of administration score Assessment of drug acceptability during administration, using a modified 6-point scale: 1 = Accepted readily, 2 = Accepted with a grimace, 3 = Accepted with a verbal complaint, 4 = Accepted with a cry, 5 = Accepted with partial loss of the drug, and 6 = Rejected entirely. Values are presented as the number of patients. Statistical analysis was performed using the Mann–Whitney U test; p < 0.05 was considered statistically significant.

Score		Oral		Nasal		P value	
								1		24		17		0.246	
2		5		4											
						3		1		2					
4		0		9											
						5		1		0					
6		4		1											
						Total		35		33					

Results of secondary outcome measures

 *Sedation Status (Sedation Score)*

 No statistically significant difference was observed between the groups (P = 0.844) (Table 5).

**Table 5:** Sedation score Evaluation of sedation level using a 5-point scale: 1 = Agitated, 2 = Alert, 3 = Calm, 4 = Drowsy, and 5 = Asleep. Values are presented as the number of patients. Statistical analysis was performed using the Mann–Whitney U test; P < 0.05 was considered statistically significant.

Score		Oral		Nasal		P-value	
								1		5		3		0.844	
2		3		3											
						3		23		24					
4		1		1											
						5		3		2					
Total		35		33											

Ease of Parental Separation (Parental Separation Score)

No statistically significant differences were observed between the groups (P = 0.995) (Table 6).

**Table 6:** Parental separation score Degree of ease with which the child separated from the caregiver: 1 = Easy separation, 2 = Whimpers but easily reassured, 3 = Cries but not clinging, and 4 = Crying and clinging to the parent. Values are presented as the number of patients. Statistical analysis was performed using the Mann–Whitney U test; P < 0.05 was considered statistically significant.

Score		Oral		Nasal		P-value	
								1		30		28		0.844	
2		2		2											
						3		0		3					
4		3		0											
						Total		35		33					

Mask Acceptance Score at Anesthesia Induction

No statistically significant differences were observed between the groups (P = 0.98) (Table 7).

**Table 7:** Mask acceptance score Child’s response to anesthesia mask placement during induction: 1 = Excellent (unafraid, cooperative), 2 = Good (slight fear, easily reassured), 3 = Fair (moderate fear, not easily reassured), 4 = Poor (terrified, crying or combative). Values are presented as the number of patients. Statistical analysis was performed using the Mann–Whitney U test; P < 0.05 was considered statistically significant.

Score		Oral		Nasal		P-value	
								1		28		24		0.98	
2		3		5											
						3		1		0					
4		5		4											
						Total		35		33					

Safety evaluation results

No serious adverse events related to midazolam premedication occurred in either group.

Furthermore, no clinical abnormalities such as respiratory depression (decreased SpO₂), anaphylactic reactions, circulatory abnormalities, delayed awakening or paradoxical responses were observed during or after surgery. In addition, no deviations from normal values were observed in blood tests (AST, ALT, Cr, BUN, etc.) conducted the day after surgery.

## Discussion

Children with physical illnesses often experience psychological and social stresses, such as anxiety and pain associated with treatment (e.g., medication, tests, and injections), and dissatisfaction with behavioral restrictions, in addition to direct physical pain caused by their illness. In particular, children with chronic physical illnesses who require long-term treatment may experience prolonged and severe psychosocial stress and exhibit pronounced stress responses [8].

Surgery under general anesthesia is a potential source of psychosocial stress. Children with a cleft lip and palate require multiple general anesthetics and surgeries, such as cleft lip and palate repair, cleft lip and nose reconstruction, and cleft palate bone grafting, until adulthood, resulting in considerable psychosocial stress. Undergoing general anesthesia and surgery while harboring such intense anxiety may lead to abnormal behavior postoperatively or delay recovery. In addition, symptoms of mental anxiety or emotional instability may persist even following discharge. Postoperative abnormal behavior generally refers to actions such as nightmares, sleep disorders, enuresis, eating disorders, depression, anxiety neurosis, or aggressive personality traits, which were not observed preoperatively and are believed to be considerably influenced by perioperative anxiety or pain.

Children with high preoperative anxiety have been reported to be at a higher risk of developing abnormal behavior postoperatively [9]. Furthermore, some studies have reported that children who have experienced hospitalization caused by surgery or illness by the age of 5, particularly those with prolonged or repeated hospital stays, are likely to develop abnormal behavior or learning disabilities during adolescence [10]. Therefore, undergoing general anesthesia and surgery while experiencing significant preoperative anxiety potentially increases the risk of abnormal behavior. To protect patients from psychological trauma caused by the highly specialized environment of surgery, it is crucial to alleviate anxiety and stress as much as possible preoperatively.

This study investigated the efficacy and safety of midazolam administered orally or intranasally as premedication in pediatric patients scheduled for cleft lip and palate surgery. The primary outcome measure, “patient response at the time of administration (Facial Hedonic Score),” exhibited a substantial tendency toward more unpleasant reactions in the intranasal administration group compared with the oral administration group. This suggests that intranasal administration is a physically and sensorially less acceptable method for younger children.

No statistically significant differences were observed between the oral and intranasal administration groups in terms of ease of administration (Ease of Administration Score), sedative effect, ease of mother-infant separation, and mask acceptance during anesthesia induction. These findings suggest that both administration routes are clinically feasible options. In this study, the Facial Hedonic Score was utilized as an indicator to assess the emotional response of patients during drug administration. The intranasal administration group exhibited significantly lower scores compared with the oral group, indicating a tendency toward stronger unpleasant reactions. This may reflect the pharmacological characteristics of midazolam, as well as the irritating and burning sensations, and physical contact with the nasal mucosa associated with intranasal administration, which is more likely to elicit psychological resistance, particularly in young children. 

Karl et al. reported that crying behavior occurred significantly more frequently in children receiving intranasal midazolam compared with the sublingual route (71% vs. 18%, P < 0.0001), suggesting greater emotional discomfort with intranasal administration [11]. Wilton et al. investigated intranasal midazolam sedation in preschool children and found it to be effective for pre-anesthetic sedation, although sensory irritation during administration was noted in some cases [6].

In summary, while intranasal administration offers superior drug absorption efficiency, care should be taken to reduce emotional stress in pediatric patients. Particularly in cases of initial surgery or children with strong anxiety tendencies, oral administration may be a more preferable option.

The Ease of Administration Score was utilized as an objective measure to assess the smoothness of medication administration techniques and the acceptance attitude of pediatric patients. Although no statistically significant differences were observed between the groups in this study, it is noteworthy that a higher proportion of cases were rated as “Score 1 (easily accepted)” in the oral administration group. This could be attributed to the following factors: (i) Many patients disliked the spray upon seeing it, as they had little to no prior experience with nasal sprays in their daily lives, (ii) the older age individuals in the intranasal administration group demonstrated greater awareness of the nonroutine nature of the procedure, and (iii) intranasal administration required spraying into both nostrils, whereas oral administration was performed as a single dose, and this difference in the number of administrations may also have affected acceptance. Among elementary school students and older children, many were able to follow instructions, but younger children were unable to follow instructions, thereby requiring restraint of the head or body, which may have increased their aversion. In addition, after spraying, the medication sometimes dripped into the throat, resulting in complaints of bitterness or spiciness. In heavier patients, administration of the undiluted solution led to a stronger bitterness, which may have been a contributing factor. Moreover, in patients with cleft palate, where part of the nasal floor is missing and communicates with the oral cavity, the medication may have entered the oral cavity, resulting in complaints of bitterness or spiciness. Meanwhile, with oral administration, the act of drinking juice is not an unfamiliar behavior, enabling patients to naturally take the medication without requiring restraint. Although individual differences exist in taste perception, the fact that the bitterness of midazolam is often not strongly perceived indicates that this is a less painful administration method for children. However, several patients demonstrated good scores with intranasal administration, and this administration method may be suitable for cooperative older children or younger children with underdeveloped swallowing reflexes owing to the rapid onset of effect via mucosal absorption. Thompson et al. reported that tolerance to taste and administration route depend on age, previous experience, and medication experience at home, requiring individualization [5]. Mennella et al. also reported that taste sensitivity in children changes with age and that tolerance to taste and administration methods is age-dependent [12]. Therefore, when selecting an administration route for premedication, decisions should be made based on a multifaceted assessment that considers not only pharmacokinetics and onset of action but also the patient’s age, psychological acceptability, and parental preferences.

This study provides clinically relevant insights by investigating the effects of the administration routes of midazolam premedication on the emotional responses and preoperative behavior of pediatric patients through a randomized controlled trial, highlighting the need for caution regarding emotional stress and limitations in acceptability associated with intranasal administration. In recent years, perioperative anxiety reduction in children has become a critical issue alongside pain management, and the role of premedication is also being reexamined [9]. McCann et al. also discussed the importance of perioperative care, including anxiety management [13], and the results of this study are significant in such a context. In Japan, the requirements for obtaining parental consent and providing information have been strengthened, increasing the importance of comfort and psychological well-being for pediatric patients. Therefore, the findings of this study are of considerable value from a policy and ethical perspective. In the absence of evidence on the standardization of premedication, prospective direct comparison studies, such as the present one, provide important basic data for the development of pediatric anesthesia guidelines and clinical decision-making. In particular, they may provide useful guidelines for case selection and optimization of the administration route for each age group.

This study has several limitations. First, the subjects’ age range was wide, from 1 to 10 years, and differences in acceptability and sedative effects depending on age may have influenced the results. The lack of age-stratified analysis is a point to consider in the result interpretation. Second, some evaluation items, such as the Facial Hedonic Score, introduced observer bias, making it difficult to completely eliminate bias. Moreover, due to the obvious differences in the administration methods (oral vs intranasal), neither participants nor investigators could be blinded to the assigned group, which may have further contributed to bias. In addition, as the study was conducted at a single institution, caution is required when generalizing the external validity of the results. In the future, more precise guidelines for dose regimen selection will be established by expanding the number of cases through multicenter collaborative studies and adding subgroup analyses by age and underlying conditions. The introduction of pediatric self-assessment scales to assess the subjective comfort of pediatric patients is also worth considering. Despite these limitations, this study represents an initial investigation that clearly compares the clinical significance and limitations of oral and intranasal premedication with midazolam in pediatric perioperative management, thereby providing important findings that will contribute to future practical applications and further research.

## Conclusions

This study investigated the efficacy and safety of midazolam as a premedication for cleft lip and palate surgery in children, comparing oral and intranasal administration routes in a prospective randomized controlled trial. As regards the patients’ reactions at the time of administration (Facial Hedonic Score), the intranasal administration group exhibited significantly more unfavorable reactions, indicating poorer acceptance and greater discomfort during drug administration, whereas no significant differences in ease of administration, sedative effect, and safety between the two groups were observed.

These results suggest that oral administration is a superior option from the perspective of patient comfort. This study highlights the need to reevaluate premedication strategies with a focus on psychological considerations for pediatric patients during the perioperative period and indicates the importance of selecting administration routes tailored to age and tolerance from the perspective of personalized medicine. While further validation through age-specific analyses and multicenter collaborative studies is warranted, this study provides valuable foundational data for the clinical application of midazolam premedication in pediatric perioperative care.
